# Nonreciprocal Spin Waves in Nanoscale Hybrid Néel–Bloch–Néel Domain Walls Detected by Scanning X‐Ray Microscopy in Perpendicular Magnetic Anisotropic Fe/Gd Multilayers

**DOI:** 10.1002/adma.202508181

**Published:** 2025-08-04

**Authors:** Ping Che, Axel J. M. Deenen, Andrea Mucchietto, Joachim Gräfe, Michael Heigl, Korbinian Baumgaertl, Markus Weigand, Michael Bechtel, Sabri Koraltan, Gisela Schütz, Dieter Suess, Manfred Albrecht, Dirk Grundler

**Affiliations:** ^1^ Laboratory of Nanoscale Magnetic Materials and Magnonics Institute of Materials (IMX) École Polytechnique Fédérale de Lausanne (EPFL) 1015 Lausanne Switzerland; ^2^ Max Planck Institute for Intelligent Systems Heisenbergstraße 3 70569 Stuttgart Germany; ^3^ Institute of Physics University of Augsburg Universitätsstrasse 1 D‐86159 Augsburg Germany; ^4^ Helmholtz‐Zentrum Berlin für Materialien und Energie Albert‐Einstein‐Straße 15 12489 Berlin Germany; ^5^ Physics of Functional Materials Faculty of Physics University of Vienna Kolingasse 14‐16 Vienna A‐1090 Austria; ^6^ Vienna Doctoral School in Physics University of Vienna Kolingasse 14‐16 A‐1090 Vienna Austria; ^7^ Research Platform MMM Mathematics‐Magnetism‐Materials University of Vienna Vienna 1090 Austria; ^8^ Institute of Electrical and Micro Engineering (IEM) École Polytechnique Fédérale de Lausanne (EPFL) 1015 Lausanne Switzerland; ^9^ Present address: Laboratoire Albert Fert CNRS, Thales Université Paris‐Saclay 91120 Palaiseau France

**Keywords:** domain walls, nonreciprocity, perpendicular magnetic anisotropy, scanning transmission X‐ray microscopy, spin waves

## Abstract

Spin wave nonreciprocity is crucial for signal processing in magnonic circuits. Domain walls (DWs) have been suggested as channels for nonreciprocal spin waves (magnons) with directional‐dependent properties. However, the experimental investigations are challenging due to the low‐damping magnetic material with DWs demanded and the nanoscale length scales involved. In this study, scanning transmission X‐ray microscopy (STXM) is used to examine coherently‐excited magnons when propagating in hybrid Néel‐Bloch‐Néel DWs in amorphous Fe/Gd multilayers with perpendicular magnetic anisotropy (PMA). Well‐ordered lattices of stripe domains and DWs are created through the integration of Cobalt nanowire arrays. Their width is measured to be δ_DW_ = (60 ± 13) nm. Near 1 GHz magnons are detected with short wavelengths down to λ = (281 ± 44) nm which were channeled in the DWs. Consistent with micromagnetic simulations, the STXM data revealed a nonreciprocal magnon band structure inside the DWs. Bloch points are identified which disrupted the phase evolution of magnons and induced different λ adjacent to these topological defects. These observations provide direct evidence of nonreciprocal spin waves within hybrid Néel–Bloch–Néel DWs in PMA materials, serving as programmable waveguides in magnonic devices with directed information flow.

## Graphical abstract

Using scanning transmission X‐ray microscopy, nonreciprocal spin‐wave behavior is revealed in real space in Fe/Gd multilayers with perpendicular magnetic anisotropy, highlighting their functionality for magnonic logic circuits operating at GHz frequencies. Topological defects disrupt phase continuity while preserving nonreciprocity, demonstrating the potential of these domain walls as programmable magnonic waveguides for directed information flow.

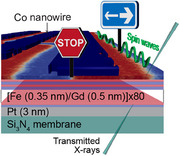

## 1 Introduction

Magnons, the quantized collective excitations of magnetic moments, can exhibit nonreciprocity in amplitude and/or frequency *f* when propagating with wave vectors **k** in opposite directions. It is of great importance for magnonic devices such as the isolators, circulators and the spin diodes.^[^ ^1^, ^2^ ^]^ Spin wave dispersion relations which are asymmetric in *k* lead to frequency nonreciprocity. This nonreciprocity can be engineered by Dzyaloshinskii–Moriya interaction (DMI),^[^ ^3^, ^4^, ^5^, ^6^, ^7^, ^8^, ^9^ ^]^ the chirality of magneto‐dipolar interactions between magnetic thin films and nanomagnets,^[^ ^10^, ^11^, ^12^, ^13^ ^]^ the curvature of a three‐dimensional nanostructure^[^ ^14^, ^15^, ^16^ ^]^ or magnetic coupling between two magnetic layers.^[^ ^17^, ^18^, ^19^ ^]^ Nonreciprocity of spin waves in domain walls (DWs) is particularly interesting for the possibility of curved short‐waved spin waves transmission. Wagner et al.^[^ ^20^ ^]^ suggested nanomagnonic circuits based on reconfigurable DWs in thin films, but relevant DW widths and spin‐wave nonreciprocity were not experimentally addressed. In ref. [21] Néel‐type DWs in a metallic ferromagnet adjacent to a heavy metal were considered and non‐reciprocal properties were predicted. However, such materials systems with interfacial DMI are known to exhibit an increased spin‐wave damping. Alternatively, Bloch‐type DWs which channel Winter‐mode spin waves^[^ ^22^, ^23^ ^]^ are expected to exhibit asymmetric dispersion relations as well. They are a result of dynamic dipolar interactions and have been modeled by micromagnetic simulations.^[^ ^24^, ^25^, ^26^ ^]^ Still, an experimental verification has so far remained elusive because DWs stabilized in a material with low damping, coherent spin wave excitation at GHz frequencies and a phase‐coherent imaging technique with a high‐spatial resolution are all required in the same setup.

In this article, we employ scanning transmission x‐ray microscopy (STXM) for imaging statically the magnetic configuration in Fe/Gd multilayers and dynamically the spin waves excited by an integrated coplanar waveguide (CPW). STXM provides a spatial resolution of 20 nm and a temporal resolution of 50 ps.^[^ ^27^, ^28^, ^29^, ^30^, ^31^, ^32^ ^]^ The amorphous Fe/Gd multilayers exhibit a perpendicular magnetic anisotropy (PMA) and low damping α of about 10^−3^.^[^ ^33^ ^]^ Different spin textures have been reported in such multilayers such as stripe domains, dipole skyrmions, and antiskyrmions if combined with Ir.^[^ ^34^, ^35^, ^36^, ^37^, ^38^ ^]^ Here, we focus on DWs which are of the hybrid Néel–Bloch–Néel type. We have prepared Cobalt (Co) nanowire arrays on top of the Fe/Gd multilayers grown on Si_3_N_4_ membrane and stabilized a preferred domain alignment. Utilizing STXM, we identify the nonreciprocal characteristics of the spin waves within the DWs excited by the torque provided by radio‐frequency (rf) currents in CPW. Depending on the frequency we observe two different signatures of nonreciprocity. At low frequencies, spin waves exhibit the same sign of the phase velocity *v*_p_ = 2π*f*/*k* (and the wave vector) but opposite sign of group velocities *v*_g_. At higher frequencies, spin waves exhibit wave vectors with different signs and magnitudes as further substantiated by micromagnetic simulations. A topological defect by a Bloch point does not modify the dispersion relation but disrupt the phase evolution of the propagating spin waves.

## 2 Results and Discussion

**Figure 1:**
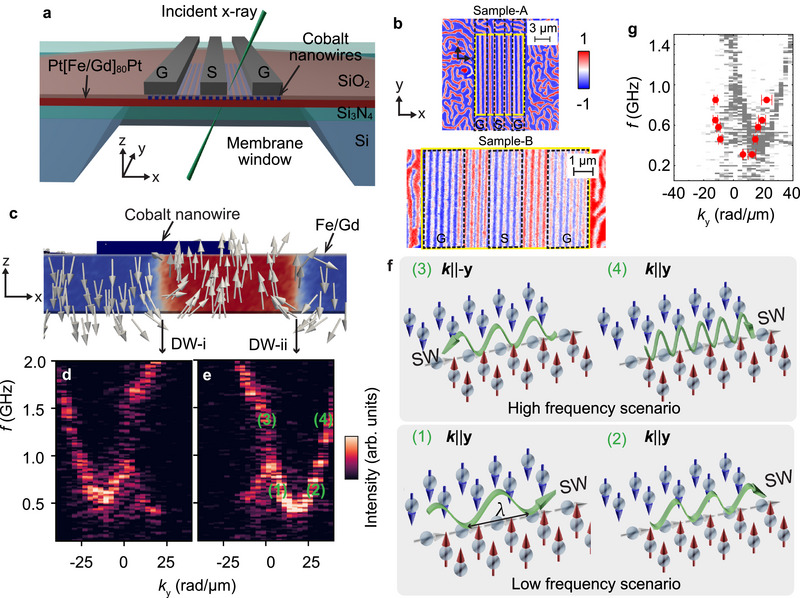
Schematic diagram and nonreciprocal spin waves channeled in the hybrid Néel–Bloch–Néel DWs formed in Fe/Gd multilayers with integrated Co nanowire arrays. a) Schematic diagram of the device and measurement configuration of STXM through the membrane window. Coplanar waveguide (CPW) consists of the ground (G) and signal (S) lines for spin wave excitation. b) Static STXM images of domains and DWs at µ_0_ *H*_⊥_ = 0 mT modified by 1D Co nanowire arrays of periodicity *p*_nw_ = 450 nm in sample‐A and *p*_nw_ = 350 nm in sample‐B. The color bar represents normalized x‐ray transmission intensity. Yellow frame indicates the region of the Co arrays and the region outside shows the disordered stripe domains in the bare Fe/Gd multilayers. Dashed black frames indicate the regions of the CPW. c) Simulated DW structure in the Fe/Gd multilayers. The red and blue colors reflect the spin orientations along the **z**‐axis. The DW‐i is underneath the Co nanowire. DW‐ii is in the gap between nanowires. d,e) Asymmetric spin wave dispersion relations corresponding to the Bloch part of the DW‐i and DW‐ii extracted from the center layer along **z**‐axis in micromagnetic simulation. f) Two scenarios of spin waves propagation with wavelength λ in the dispersion relations depicted in e). Low frequency scenario consists of (1) and (2). High frequency scenario consists of (3) and (4). Green arrows describe the phase velocity directions. g) Dispersion relations extracted from the STXM imaged spin waves within the DWs located in the gap of nanowires like a DW‐ii. The simulated dispersion relation displayed in e) is reprinted in gray color for comparison.

The cross section of the device is sketch in **Figure** **1**a. Co nanowire arrays and coplanar waveguide (CPW) consisting of signal line (S) and ground (G) lines were fabricated on amorphous [Gd(0.5 nm)/Fe(0.35 nm)]x80 multilayers and the sample preparation can be found in the Experimental Section. The magnetic force microscopy and ferromagnetic resonance data are displayed in Section S1 and Figure S1 (Supporting Information). Two samples A and B containing multiple devices will be discussed. We performed static and dynamic X‐ray circular dichroism (XMCD) measurements using the STXM at MAXYMUS endstation, Bessy II, Berlin.^[^ ^39^ ^]^ The devices were tilted to 30 degree with respect to the incident X‐ray as shown in Figure 1a in order to collect both the in‐plane and out‐of‐plane dynamic magnetization. A converting factor 2/$\sqrt{3}$ was applied to obtain the actual **x**‐axis pixels. Before performing the STXM measurements, the Co nanowires were magnetized by an in‐plane magnetic field along their long axes and then the samples were demagnetized with an oscillating out‐of‐plane field. A field µ_0_ **H**_⊥_ was applied perpendicular to the sample surface to stabilize the investigated magnetic configuration. Parameters of the STXM measurements and the data processing are described in the Experimental Section.

The static STXM image of sample‐A containing a nanowire array with stripes of periodicity *p*_nw_ = 450 nm and a width of *w*_nw_ = 225 nm is depicted in Figure 1b. The external field is 0 mT. The normalized transmitted X‐ray signal is displayed to imitate *m*_z_/*M*_S_. The integrated CPW (marked by dashed black frames Figure 1b) modified locally the magnetic contrast because it absorbed part of the X‐ray and reduced the transmission. The well‐aligned stripe domains and DWs were near the Co nanowire arrays. Here, the periodicity of the domain lattice *p*_d_ is 2*p*_nw_. The bare Fe/Gd outside the nanowire region contained the random domains. In sample‐B with slightly different materials parameters, denser domain lattices were produced which fulfilled *p*_d_ = *p*_nw_ = 350 nm (Figure 1b). STXM images of devices with different nanowire sizes on sample‐B in the Figure S2 (Supporting Information)^[^ ^40^ ^]^ showed an evolution of magnetic states from partially aligned domains to domains fully aligned with nanowires.

Micromagnetic simulations were performed using the Mumax3 code^[^ ^41^, ^42^, ^43^, ^44^ ^]^ to understand the magnetic domain structures and their spin dynamics. The static and dynamic simulation parameters are described in Experimental Section. PMA and dipole interaction cooperated to construct the DWs in Fe/Gd multilayers and the spin textures at top and bottom surfaces experienced Néel‐type rotation while the central part was Bloch‐type (Figure 1c). Later, we will distinguish two kinds of DWs considering the surrounding domains extending into *y*‐direction: spin‐down on the left and spin‐up on the right (labeled as DW‐i), and spin‐up on the left and spin‐down on the right (labeled as DW‐ii). Figure 1d,e describes the simulated asymmetric spin wave dispersion relations inside the Bloch‐type regions of these two DWs. The variation of the dispersion relations over the thickness are displayed in the Figure S3 (Supporting Information).^[^ ^40^ ^]^ The dispersion relation of the Bloch part in center (layer Nz = 10) and Néel part on the surfaces (layer Nz = 1 and 18) are consistent in terms of nonreciprocity. There are two typical scenarios of nonreciprocity in each dispersion relation. They are illustrated in Figure 1f considering the DW‐ii and the dispersion relation *f*(*k*_*y*_) shown in Figure 1e. At low frequency, spin wave excitation occurs exclusively when *k*_*y*_ > 0. The phase velocity *v*_p_ is always positive. Two modes are excited at the same time, (1) one at small *k* with *v*_g_ < 0 and (2) one at large *k* with *v*_g_ > 0. At high frequency, modes exist with *k*_*y*_ > 0 and *k*_*y*_ ⩽ 0. Here, mode (3) at *k*_*y*_ ⩽ 0 has *v*_g_ < 0, while mode (4) at *k*_*y*_ > 0 has *v*_g_ > 0. Our simulation results are consistent with the modeling and simulations performed on hybrid Néel–Bloch–Néel DWs in Refs. [24, 25]. The nonreciprocity can be understood as a consequence of dynamic dipolar fields of propagating spin waves. In Ref. [46] the authors have shown that the nonreciprocity can be derived from a non‐zero toroidal moment of a spin structure hosting the spin wave. We have computed the toroidal moment along the spin‐wave propagation direction from the simulated domain‐wall spin structure (i.e., along the Néel–Bloch–Néel domain wall) and confirmed that it was non‐zero and collinear with the spin‐wave wave vector.

**Figure 2:**
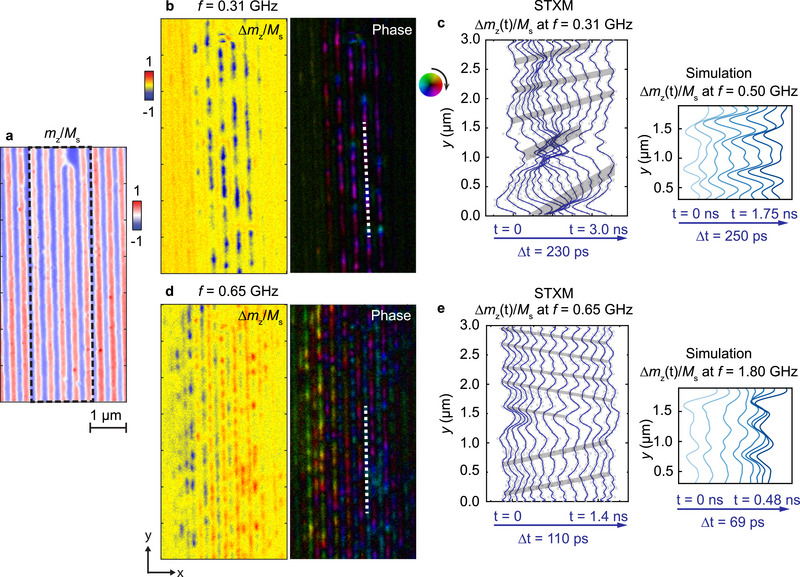
Nonreciprocal spin waves channeled in the hybrid Néel‐Bloch‐Néel DW. a) Static STXM images of the densest domains formed in Fe/Gd multilayers underneath and near the signal line in sample‐B. Here 1D Co nanowire array of periodicity *p*_nw_ = 350 nm at µ_0_ *H*_⊥_ = 0 mT is integrated. Dashed black frame indicates the region covered by the signal line. b,d) Snapshot of the spin dynamics and their phase (amplitude encoded as brightness and the phase as color) at *f* = 0.31 GHz and *f* = 0.65 GHz normalized to the static image. c,e) The time evolution of the dynamic components of the transmission signal taken from the region marked by the dashed white lines in all the phase images. Grey shadows are the eye‐guide by marking the maxima moving with time. The dynamic components shown on the right are extracted at different times from the simulations when continuously exciting at frequencies that yield wavelengths comparable to the STXM data.

**Figure** **2** shows STXM data obtained on domains in device2 of sample‐B underneath and near the signal line. In Figure 2a the regular stripe‐domain patterns is shown which is formed below the Co nanowire array with *p*_nw_ = 350 nm. The domains are about 120 nm wide and appear in red and blue color indicating regions with out‐of‐plane magnetization vectors pointing up and down, respectively. The DWs are white indicating in‐plane magnetization vectors with width of (60 ± 13) nm. We applied continuous‐wave rf currents at multiple frequencies and excited the depicted domain pattern. The dynamic magnetization Δ*m*_*z*_(*y*) (spin‐precessional amplitude) was stroboscopically detected with high temporal resolution using a pulsed x‐ray beam. In Figure 2b, a snapshot of the normalized spin‐precessional amplitudes at *f* = 0.31 GHz and the local phases (phase map) are plotted. Attributable to the DW alignment, the signal in the DW marked by the dashed white line is further examined in Figure 2c where we show the time evolution of the dynamic magnetization Δ*m*_*z*_(*y*)/*M*_s_ as a waterfall plot. The grey bars highlight how local maxima in Δ*m*_*z*_(*y*)/*M*_s_ shift in space with time. They hence define the local wavelength. It is notable that below and above *y* = 1.5 µm, these maxima show both a different separation in *y* and different temporal shift. Below (above) *y* = 1.5 µm we extract a wavelength λ_1_ = (988 ± 26) nm [λ_2_ = (504 ± 105) nm] with *v*_p_ > 0, propagating along the +*y*‐direction (Movie S1, Supporting Information). The observation agrees with the low frequency scenario illustrated in Figure 1f.

The spin dynamics of the same DW at an increased excitation frequency of *f* = 0.65 GHz is analyzed in Figure 2d,e. Below [above] *y* = 1.5 µm we now extract a wavelength λ_1_ = (515 ± 66) nm with *v*_p_ < 0 [λ_2_ = (327 ± 41) nm with *v*_p_ > 0]. The two spin waves propagate into opposite directions along the *y*‐axis (Movie S2, Supporting Information), consistent with the high frequency scenario in Figure 1f. Further STXM images at *f* = 0.46 GHz and 0.58 GHz are shown in Supplementary Materials Figure S4.^[^ ^40^ ^]^ In Figure 1g, we summarize the observed wavelengths and phase velocities. Short‐waved spin waves down to λ = (281 ± 44) nm were channeled in the DWs at *f* = 0.85 GHz. This value is more than a factor of 10^6^ times shorter than the wavelength λ_em_ of the corresponding electromagnetic wave in free space and, to our knowledge, a record on‐chip miniaturization of λ_em_. It substantiates the prospects of nanomagnonics based on domain walls.^[^ ^20^, ^45^, ^47^, ^48^, ^49^ ^]^ The experimental data provide the dispersion relation *f*(*k*_*y*_) inside the investigated DW. We find a clear asymmetry between the spin wave branches at positive and negative wave vectors *k*_*y*_. The experimentally resolved nonreciprocity for spin waves in the DW agrees qualitatively well with the characteristics extracted from micromagnetic simulations (Figure 1e). The remaining discrepancy concerning measured and simulated eigen‐frequencies might be caused by the modification of the magnetic properties of the Fe/Gd multilayers during the nanofabrication. The lithography for lift‐off processing of Co nanowires and CPWs involved processes at elevated temperatures which facilitated partial interdiffusion of Fe and Gd. This effect may have led to modifications in the PMA strength and saturation magnetization, resulting in a decrease in nonreciprocity in the high‐frequency regime and an enhancement in the low‐frequency regime, as shown in Figure 1g.

**Figure 3:**
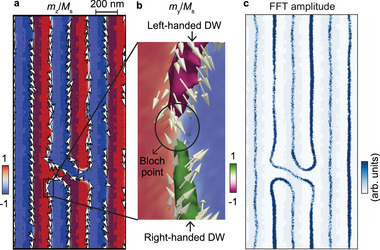
Micromagnetic simulations revealing the well‐aligned DWs formation and their asymmetric dynamic features. a) Static magnetization *m*_z_/*M*_S_ of the domain configuration extracted from micromagnetic simulations in the layer number 12 along z‐direction of Fe/Gd film. White arrows with black outline reflect the in‐plane magnetization directions. b) Zoom of a) with isosurfaces of *m*_y_/*M*_S_ = ± 0.75, showcasing domain walls with opposite handedness separated by a Bloch point. The color bar indicates *m*_z_/*M*_S_. c) Simulated spin dynamics amplitude at *f* = 0.42 GHz. The legend indicates the logarithmic scale of the normalized Fourier component of the magnetization in resonance. Blue shadows in a) and c) indicate the Co nanowires with defects.

Near *y* = 1.5 µm in Figure 2c,e, a spin pinning effect affects the spin dynamics. To gain microscopic insight, we carried out dynamic simulations for a DW containing a Bloch point (black rectangle in **Figure** **3**a). Near this point, the in‐plane magnetization vectors rotate by 180 degrees (Figure 3b). As a consequence, the handedness of the DW switches locally. This variation cannot explain the two different spin waves above and below *y* = 1.5 µm as the handedness does not modify the dynamic dipolar interaction; the dispersion relation in the Bloch‐type DW stays the same.^[^ ^25^ ^]^ We assume that the non‐collinear spin structure at the Bloch point operates as a point‐like scatterer upon microwave excitation allowing us to emit spin waves propagating in opposite directions inside the DW at high frequency. It is notable that the Bloch point is unstable and can be annihilated during micromagnetic simulations, but the dispersion relations around the Bloch point remain consistent in all simulations, as long as the Bloch point persists.

In Figure 2 and Figure S4 (Supporting Information) we observed that at the small frequencies *f* = 0.31 GHz and 0.46 GHz not all DWs were excited. Instead, spin waves existed particularly in the DWs‐ii. The same behavior was reproduced by our micromagnetic simulations (Figure 3c). This is different for the higher excitation frequencies for which both DWs‐ii and DWs‐i hosted spin waves. At *f* = 0.58 GHz, two spin waves modes of λ = (739 ± 85) nm and λ = (803 ± 141) nm with *v*_p_ < 0 were detected by STXM, consistent with the low frequency scenario in the dispersion relation shown in Figure 1e. This observation indicated that the dispersion relation in DWs‐i was blue shifted compared to DWs‐ii. We noted that this discrepancy existed underneath the Co nanowire arrays and vanished for DWs in a bare Fe/Gd multilayer as shown in the dynamic STXM images of Figure S5 (Supporting Information). In bare Fe/Gd, spin waves were channeled in both kinds of DWs which corroborated that the Co nanowires introduced the frequency‐dependent selection rule. The incorporation of the Co grating influences the magnons in the underlying Fe/Gd in two ways: first, by inducing a shift in resonance frequencies due to a stray field modifying the effective magnetic field (static coupling); and second, by altering the magnon bands in a wavevector‐dependent manner through dynamic dipolar interaction with Co grating (dynamic coupling). To distinguish between static and dynamic dipole contributions, we investigated the dynamic response of the DWs with the Co magnetization fixed with micromagnetic simulation, where the dynamic dipole coupling is turned off (Figure S6, Supporting Information). In this scenario, the dispersion relations of magnons in both DW‐i (under Co) the DW‐ii (in gap of the nanowires) are modified. The dispersion of DW‐i shifts significantly upward by approximately 1 GHz, while the additional branch in DW‐ii disappears. The shift in DW‐i can be attributed to the static coupling that the stray field generated by the Co is dominating. Conversely, the dynamic dipole fields from the precessing Fe/Gd magnetization can couple with forced precessional modes in Co, causing a shift for *k* ≠ 0. Meanwhile, dipole‐mediated coupling can occur between the DWs. In Figure 1d,e, both the dispersion in DWs underneath the Co and in gap of the nanowires show the appearance of another branch at *k* = 0 which vanishes at higher *k*. This mode arises from dipole‐mediated coupling between DWs, with coupling strength decreasing as *k* increases. This effect is evident in the low‐frequency *k* = 0 dynamic amplitudes, which exhibit an acoustical mode consistent with an array of coupled DWs.^[^ ^25^ ^]^

**Figure 4:**
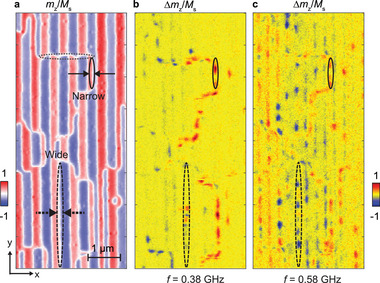
Super‐domain formation and the dynamic features. a) Static STXM image of domains formed by Co nanowire arrays with *p*_nw_ = 300 nm. Solid black arrows and oval mark the example of a narrow DW. Dashed black arrows and oval mark wide DW. Dotted gray oval marks the example of DW segments along x‐direction. b,c) Snapshots of the DWs dynamics at *f* = 0.38 GHz and *f* = 0.58 GHz, respectively.

In **Figure** **4**a, we show an STXM image taken on an Fe/Gd multilayer underneath a Co nanowire array with a small periodicity *p*_nw_ = 300 nm (sample‐B device1). We observe a particular domain configuration with different types of DWs, which we call super‐domain structure in the following. Considering Figure S7 (Supporting Information), the so‐defined super‐domain structure consists of distinct regions (super‐domains) separated by curved boundaries (black lines in Figure S7, Supporting Information). At the curved boundaries the phase of the stripe‐shaped pattern of the individual domains (indicated by blue and red color) shifts. Inside a super‐domain the periodic pattern exhibits a periodicity which is twice that of the Co stripe periodicity *p*_nw_. The majority of individual domains have a width of about 220 nm. Between such domains we find a DW along *y*‐direction (dotted black oval in Figure 4a) which we label wide‐domain DW (w‐DW). Its width amounts to (83 ± 21) nm. The combined width of an individual domain and a w‐DW agrees with with *p*_nw_ in such a pattern. A few domains are locally narrower than 220 nm as additional DWs exist, highlighted by the solid black oval in Figure 4a and labeled narrow‐domain DW (n‐DW). We find that the n‐DWs are located in gaps between Co nanowires. Their width amounts to (52 ± 10) nm. Furthermore, there are DW segments which extend along the *x*‐direction as what is marked by gray dotted oval in Figure 4a. Their width amounts to (104 ± 17) nm. They connect to both w‐DWs and n‐DWs and thereby produce the super‐domain structure consisting of patches of regular patterns of individual 220‐nm‐wide stripe domains separated by w‐DWs. Figure 4b displays a snapshot of the dynamic STXM images taken for an excitation frequency *f* = 0.38 GHz. Prominent spin‐precessional motion (dark red and dark blue) is found only in a few narrow channels which follow the curved boundaries defining the super‐domain structure. Inside the patches of regular 300‐nm‐wide domains the excitation is weak. A similar distribution of spin‐precessional amplitudes is found at *f* = 0.46 GHz (not shown). The detailed analysis reveals that channels along the *y*‐axis host propagating spin waves, while along the *x*‐axis standing spin waves exist. At *f* = 0.58 GHz displayed in Figure 4c, the distribution of spin‐precessional amplitudes is found to be completely different. Spin waves are now channeled inbetween many more wide and narrow domains. In the dashed black oval, we find two propagating spin wave modes with *v*_p_ > 0 which exhibit wavelengths λ = (672 ± 80) nm and λ = (395 ± 72) nm in w‐DW. The observation is consistent with the low‐frequency scenario of Figure 1f. The experimental data obtained on sample‐B device1 further corroborate the nonreciprocal spin wave dispersion relations inside DWs in Fe/Gd multilayers. We note that spin waves propagating along the *y*‐direction are counter‐intuitive as their wave vectors are collinear with the direction of the rf current in the CPW. We assume that a defect or again a Bloch point inside the DW allowed us to emit spin waves into *y*‐direction.

## 3 Conclusion 

In summary, we presented the experimental exploration of nonreciprocal spin wave dispersion relations in hybrid Néel‐Bloch‐Néel DWs in Fe/Gd multilayers by dynamic STXM measurements. The precisely engineered Fe/Gd multilayers with PMA and Co nanowire arrays facilitated the formation of complex DW configurations, within which spin waves were channeled and exhibited distinct nonreciprocal behaviors. We demonstrated that the presence of topological singularities known as Bloch points did not alter the nonreciprocity of spin waves. However, they affected their phases. Depending on the excitation frequency, we realized unidirectional flow of spin waves exhibiting two characteristic wavelengths determined by the nonreciprocal spin‐wave dispersion relation. Our experiments indicate that integrated nanomagnets can be used to imprint super‐domain structures and complex DW configurations which channel spin waves through Fe/Gd multilayers. The minimum channel width amounts to about 50 nm. Compared with top‐down fabricated waveguides which confine magnon modes between edges with unintentional roughness,^[^ ^50^, ^51^, ^52^ ^]^ the nanoscale DWs have three key advantages: they allow for the spontaneous excitation of short‐wavelength spin waves comparable to vortices considered in Refs. [48, 49]; they have uniform magnetization for spin‐wave propagation such that spin waves can follow adiabatically a curved trajectory;^[^ ^23^ ^]^ and they are not affected by edge roughness from nanofabrication. **Table** **1** summarizes key characteristics and benefits of different kinds of realized and proposed spin‐wave channels based on magnetic textures and nanofabricated magnon waveguides. We simulated programmable magnonic logic gates consisting of two parallel hybrid Néel–Bloch–Néel DWs in a Fe/Gd multilayer. Thereby, we explored interfering spin wave signals for the implementation of NOT, OR and XOR gates and present the simulation results in Section S8 and Figure S8 (Supporting Information).

**Table 1:** Comparison of spin wave channels, their materials and format, damping parameter α, spin‐wave wavelength λ and frequency *f*, conversion factor *CF*, and further benefits.

Spin‐wave	Material	α	λ [nm]	*CF* ^a)^	Benefit^b)^	Refs.
channel	and Format	[10^−3^]	(*f* [GHz])	[10^5^]		
Hybrid Néel‐Bloch‐Néel DWs	Fe/Gd layers with PMA between Pt films	9	504 (0.31)	19.2	1,2,3,4,5	t.w.
Bloch‐type DWs	Pt/Co/Al_2_O_3_	10^c)^	∼60 (10)	5	1,2,3,4,5	[21]
Mixed Néel‐ and Bloch‐type DWs	Ni_81_Fe_19_/Ru/Co_40_Fe_40_B_20_	8^c)^	86 (1.11)	7	1,2,4,5	[23]
Néel‐type DWs	Ni_81_Fe_19_	7^c)^	6280 (0.2)	2.4	2,4,5	[20]
Interfacial DMI formed Néel‐type DWs	Gd_23_Fe_67.4_Co_9.6_	5^c)^	∼165 (30)	0.61	2,3,4,5	[55]
Bloch‐type Skyrmion tubes	Cu_2_OSeO_3_ platelet (at *T* = 25 K)	8	12000 (1 to 6)	0.42	2,3	[54]
DW in Synthetic Antiferromagnet	Co_40_Fe_40_B_20_/Ru/Co_40_Fe_40_B_20_/Ir_22_Mn_78_/Ru	4	340 (1.43)	6.2	2,3,4,5	[31]
	Co_4_					
Strip or Disk made by micro‐ and nanofabrication	Ni_80_Fe_20_ microstrip	10^c)^	1750 (1–6)	1.71	5	[53]
	Ni_81_Fe_19_ microstrip	–	6000 (2.75)	0.18	5	[50]
	Y_3_Fe_5_O_12_ nanostrip	0.21	∼2000 (∼3.5)	0.43	6,7	[51]
	Y_3_Fe_5_O_12_ nanostrip	0.18	600 (3.78)	1.32	6,8	[52]
	Co/Ru/Ni_81_Fe_19_ disk	10^c)^	5000 (0.5)	1.2	3,4,5	[57]

^a)^ Conversion Factor *CF* = (Wavelength of the free‐space microwave at *f*)/(Wavelength λ of the spin wave);

^b)^ Benefit: 1. Spin waves follow adiabatically a curved trajectory; 2. Not affected by edge roughness from nanofabrication; 3. Nonreciprocal spin‐wave dispersion relations; 4. Internal short‐wave magnon emitter demonstrated; 5. Compatible to the standard semiconductor technology; 6. Low damping for nonlinear effects; 7. Coupling between nanochannels for half‐adders; 8. Ultrabroad bandwidth possible for multifrequency applications;

^c)^ Parameter used for simulation.

t.w.: This work.

The hybrid Néel–Bloch–Néel domain walls discussed in our work realize a more pronounced miniaturization of the corresponding electromagnetic wave than anticipated before and exhibit nonreciprocity that allows for functional magnonic devices.^[^ ^53^ ^]^ Compared to other material systems hosting nonreciprocal spin waves, such as chiral magnets with skyrmion tubes,^[^ ^54^ ^]^ interfacial‐DMI‐stabilized Néel‐type DWs,^[^ ^55^ ^]^ and synthetic antiferromagnets (SAFs) that show anisotropic spin wave properties,^[^ ^31^, ^56^, ^57^ ^]^ hybrid Néel–Bloch–Néel DWs in Fe/Gd multilayers provide low‐damping magnonic waveguides that operate at room temperature and can guide spin waves isotropically along curved trajectories. Our findings pave the way for the design of magnonic logic circuits at GHz frequencies which make use of nonreciprocal magnon band structures in ultra‐narrow spin‐wave channels formed by DWs in PMA thin films.

## 4 Experimental Section

### Sample Description and Preparation

The amorphous Fe/Gd multilayers were grown in a ultra high vacuum (UHV) environment under 3.5 µbar argon pressure at room temperature by DC‐magnetron sputtering on 100 nm‐thick Si_3_N_4_ membranes, to enable X‐ray transmission in scanning transmission X‐ray microscopy (STXM). Ultra‐thin Gd and Fe layers were deposited alternatively for multiple times, i.e., 0.5 nm‐thick Gd and 0.35 nm‐thick Fe for 80 times. The stack was capsuled by 3 nm‐thick Platinum layers on the bottom and top, acting as seed and cover layer, respectively. Co nanowire arrays with multiple widths and periodicity were defined by electron beam lithography directly on the Pt/[Fe/Gd]_80_/Pt multilayers. Their lateral sizes are listed in the Table S1 (Supporting Information). The evaporated Co nanowires are 20 nm thick. Coplanar waveguides (CPW) made of Al (120 nm)/Cu (10nm) with 2.1 µm‐wide ground lines (G) and a 1.6 µm‐wide signal line (S) were integrated for spin wave excitation. A thin layer of SiO_2_ was placed between the nanowires and the CPW for electrical insulation.

### Time‐Resolved Scanning Transmission X‐Ray Microscopy and data Processing

Synchrotron‐based time‐resolved STXM (TR‐STXM) was conducted at the MAXYMUS endstation at BESSY2, HZB, Berlin, Germany.^[^ ^39^ ^]^ The monochromatic X‐rays were focused using a zone plate, and 2D spatial maps such as Figures 1b, 2a, and 4a were generated through point‐to‐point scans. Scanning step of 25 nm was used for the static and dynamic scans shown in Figure 1b lower panel, Figures 2 and 4, and Figures S2, S4 and S5 (Supporting Information) and 88 nm for the static scan in Figure 1b upper panel. The scanning stages give the positioning accuracy better than 2 nm, so that the spatial resolution is determined by the X‐ray optics. The circularly polarized X‐rays at the Gd M5 edge, 1187.6 eV was incident to the membrane window and the transmitted X‐rays counts are collected. In this configuration, XMCD is sensitive to the magnetic components parallel to the X‐ray incident direction.^[^ ^58^ ^]^ Thus, to collect both the in‐plane and out‐of‐plane components of the dynamic magnetic precession in the Fe/Gd multilayers with perpendicular magnetic anisotropy, we tilted the sample to 30 degree with respect to the X‐ray incident angle. A converting factor 2/$\sqrt{3}$ was applied to obtain the actual **x**‐axis pixels. Magnetic field was applied via a well‐calibrated system consisting of four permanent magnets. When the sample is tilted, we rotate the magnetic field so that it is perpendicular to the membrane window to maintain the magnetic phase diagram.

Time resolution was achieved through stroboscopic imaging using X‐ray pulses. TR‐STXM in multi‐bunch operation mode at Bessy II utilizes the continuous RF current as the pump and pulsed synchrotron flashes with a 60 ps width and a 2 ns periodicity as probes.^[^ ^39^ ^]^ In this mode, the temporal resolution is about 100 ps. Under the specialized low‐alpha synchrotron operation mode, this can be extended down to 10 ps and TR‐STXM can operate at frequencies up to 50 GHz.^[^ ^59^, ^60^ ^]^ We utilized multi‐bunch modes and 13 channels in one periodicity for all the investigated frequencies. To study the dynamic magnetic response, a continuous rf current was applied to the S‐line in CPW (inductive rf currents were generated in the G‐lines), and measurements were conducted using the pulsed X‐ray beam with an effective width of approximately 100–200 ns. We collected 13 temporal channels in one periodicity for all the frequencies. With the point‐to‐point scan for 13 channels, raw TR‐STXM data were collected and a time‐averaging normalization for a selected channel denotes the Δ*m*_*z*_/*M*_*S*_ as shown in Figure 2b,d as well as Figure 4b,c. By this method, we remove the photon flux variations coming from the X‐ray endstation electronics. Dwell time of 40 ms was set for data collection of each point. A Fourier filter was further applied to remove noises synchronized with the excitation frequencies.^[^ ^61^ ^]^ The color map in Figure 2b,d shows also the relative phase of the spin waves with amplitude encoded as brightness and the phase as color, via a temporal fast Fourier transform algorithm.

### Micromagnetic Simulation Parameters

Micromagnetic simulations were performed using the Mumax3 code.^[^ ^41^ ^]^ The Fe/Gd multilayers were simulated as a 68 nm thick effective material with magnetic parameters based on the magnetic force microscope and ferromagnetic resonance characterizations (Section S1, Supporting Information). Simulations were conducted using a temperature of *T* = 300 K.^[^ ^42^ ^]^ We note that gives a mesh dependence to the results.^[^ ^43^ ^]^ We use a saturation magnetization *M*_S, FeGd_ = 320 kA m^−1^, exchange stiffness *A*_FeGd_ = 6 pJ m^−1^, gryomagnetic ratio |γ| = 176 rad GHz T^−1^ and out‐of‐plane uniaxial anisotropy *K*_U_ = 66 kJ m^−3^. On top of the Fe/Gd a 18.3 nm thick Co grating was placed with periodicity *p*_nw_ = 325 nm and width *w*_nw_ = 162 nm. The interlayer exchange coupling between Co and Fe/Gd is deactivated due to the covering 3 nm‐thick Pt layer. Randomly distributed indentations were made along the width of the Co grating to account for defects present in the fabrication. For the Co nanowires parameters, the saturation magnetization *M*_S, Co_ = 1440 kA m^−1^, exchange stiffness *A*_Co_ = 30 pJ m^−1^, gyromagnetic ratio |γ| = 176 rad GHz T^−1^, and Gilbert damping α_Co_ = 0.5 were utilized.^[^ ^62^ ^]^ The system was discretized into 256 × 512 × 24 cells of dimensions 3.8 × 4 × 3.7 nm^3^. 10 repetitions of periodic boundaries were applied along the **x**‐ and **y**‐directions.^[^ ^44^ ^]^

The system was initialized similarly to the experiment. First, a field of 200 mT was applied along the *y*‐axis and the system was relaxed by running the simulation for 10 ns using the Dormand‐Prince method. Then, the in‐plane field was removed and the system was demagnetized with an oscillating out‐of‐plane field. The resulting remanent state was used as an initial state for the dynamic simulations.

To compute the dispersion, an oscillating magnetic field pulse of the form $h_{0}\mathrm{sinc}\left(2\pi f_{c}\left(t-T\right)\right)$ with amplitude *µ*_0_ *h*_0_ = 3 mT, cut‐off frequency *f*_c_ = 3 GHz and time‐offset *T* = 33 ns was applied to a central region of width 20 nm along 45 degree rotated from **x**‐axis in the **x**‐**z** plane. The width is chosen to efficiently excite spin wave vectors in relevant range as obtained in the STXM experiments. The simulations were run for 67 ns and the magnetization was sampled every 167 ps. To improve the quality of the dispersion, the damping of the Fe/Gd was set to α = 10^−5^. Absorbing boundary conditions were applied along the *y*‐axis^[^ ^44^ ^]^ and the periodic boundary conditions of the static simulation were maintained. A 2D fast Fourier transform (FFT) was performed on the dynamic *z*‐component of the magnetization along the domain walls, yielding the dispersion shown in Figure 1e,f.

The amplitude map of Figure 3c was obtained in the following manner. First, a spatially homogeneous sinc pulse with the same time dependence as outlined above was used to excite the system. Following the same procedure, a 1D FFT was performed on the dynamic magnetization, and the modulus of the resulting magnetization was averaged over the central Fe/Gd layer. From this, resonance frequencies were extracted. Figure 3c shows the modulus of the complex dynamic magnetization at resonance frequency *f* = 0.42 GHz.

## Statistical Analysis

Time‐resolved dynamic magnetization data from both X‐ray experiments and simulations were processed using normalization to the static magnetization. Data are presented as mean ± standard deviation (SD) where applicable. Sample sizes, devices, and scan areas are stated in the figure legends. Data analysis was performed using Python, MATLAB and OriginPro.

## Conflict of Interest

The authors declare no conflict of interest.

## Supporting information

Supporting Information

Supplemental Movie 1

Supplemental Movie 2

## Data Availability

The data that support the findings of this study are available from the corresponding author upon reasonable request.

## References

[1] A. V. Chumak , P. Kabos , M. Wu , C. Abert , C. Adelmann , A. O. Adeyeye , J. Åkerman , F. G. Aliev , A. Anane , A. Awad , C. H. Back , A. Barman , G. E. W. Bauer , M. Becherer , E. N. Beginin , V. A. S. V. Bittencourt , Y. M. Blanter , P. Bortolotti , I. Boventer , D. A. Bozhko , S. A. Bunyaev , J. J. Carmiggelt , R. R. Cheenikundil , F. Ciubotaru , S. Cotofana , G. Csaba , O. V. Dobrovolskiy , C. Dubs , M. Elyasi , K. G. Fripp , et al., IEEE Trans. Magn. 2022, 58, 1.

[2] B. Flebus , D. Grundler , B. Rana , Y. Otani , I. Barsukov , A. Barman , G. Gubbiotti , P. Landeros , J. Akerman , U. Ebels , P. Pirro , V. E. Demidov , K. Schultheiss , G. Csaba , Q. Wang , F. Ciubotaru , D. E. Nikonov , P. Che , R. Hertel , T. Ono , D. Afanasiev , J. Mentink , T. Rasing , B. Hillebrands , S. V. Kusminskiy , W. Zhang , C. R. Du , A. Finco , T. van der Sar , Y. K. Luo , et al., J. Phys. Condens. Matter 2024, 36, 363501. 10.1088/1361-648X/ad399c 38565125

[3] J. Moon , S. Seo , K. Lee , K. Kim , J. Ryu , H. Lee , R. D. McMichael , M. D. Stiles , Phys. Rev. B 2013, 88, 184404.

[4] K. Di , V. L. Zhang , H. S. Lim , S. C. Ng , M. H. Kuok , J. Yu , J. Yoon , X. Qiu , H. Yang , Phys. Rev. Lett. 2015, 114, 047201. 25679905 10.1103/PhysRevLett.114.047201

[5] M. Küß , M. Hassan , Y. Kunz , A. Hörner , M. Weiler , M. Albrecht , Phys. Rev. B 2023, 107, 024424.

[6] D. Cortés‐Ortuño , P. Landeros , J. Phys. Condens. Matter 2013, 25, 156001. 23507871 10.1088/0953-8984/25/15/156001

[7] S. Seki , Y. Okamura , K. Kondou , K. Shibata , M. Kubota , R. Takagi , F. Kagawa , M. Kawasaki , G. Tatara , Y. Otani , Y. Tokura , Phys. Rev. B 2016, 93, 235131.

[8] T. J. Sato , D. Okuyama , T. Hong , A. Kikkawa , Y. Taguchi , T. Arima , Y. Tokura , Phys. Rev. B 2016, 94, 144420.

[9] P. Che , I. Stasinopoulos , A. Mucchietto , J. Li , H. Berger , A. Bauer , C. Pfleiderer , D. Grundler , Phys. Rev. Res. 2021, 3, 033104.

[10] C. Liu , J. Chen , T. Liu , F. Heimbach , H. Yu , Y. Xiao , J. Hu , M. Liu , H. Chang , T. Stueckler , S. Tu , Y. Zhang , Y. Zhang , P. Gao , Z. Liao , D. Yu , K. Xia , N. Lei , W. Zhao , M. Wu , Nat. Commun. 2018, 9, 738. 29467416 10.1038/s41467-018-03199-8 PMC5821877

[11] J. Chen , T. Yu , C. Liu , M. Madami , K. Shen , J. Zhang , S. Tu , M. S. Alam , K. Xia , M. Wu , G. Gubbiotti , Y. M. Blanter , G. E. W. Bauer , H. Yu , Phys. Rev. B 2019, 100, 104427.

[12] H. Wang , J. Chen , T. Yu , C. Liu , C. Guo , S. Liu , K. Shen , H. Jia , T. Liu , J. Zhang , M. A. Cabero , Q. Song , S. Tu , M. Wu , X. Han , K. Xia , D. Yu , G. E. W. Bauer , H. Yu , Nano Res. 2021, 14, 2133.

[13] L. Temdie , V. Castel , C. Dubs , G. Pradhan , J. Solano , H. Majjad , R. Bernard , Y. Henry , M. Bailleul , V. Vlaminck , AIP Adv. 2023, 13, 025207.

[14] R. Hertel , SPIN 2013, 03, 1340009.

[15] J. A. Otálora , M. Yan , H. Schultheiss , R. Hertel , A. Kákay , Phys. Rev. Lett. 2016, 117, 227203. 27925729 10.1103/PhysRevLett.117.227203

[16] M. C. Giordano , M. Hamdi , A. Mucchietto , D. Grundler , Phys. Rev. Mater. 2023, 7, 024405.

[17] R. A. Gallardo , T. Schneider , A. K. Chaurasiya , A. Oelschlägel , S. S. P. K. Arekapudi , A. Roldán‐Molina , R. Hübner , K. Lenz , A. Barman , J. Fassbender , J. Lindner , O. Hellwig , P. Landeros , Phys. Rev. Appl. 2019, 12, 034012.

[18] M. Ishibashi , Y. Shiota , T. Li , S. Funada , T. Moriyama , T. Ono , Sci. Adv. 2020, 6, 17. 10.1126/sciadv.aaz6931 PMC7182415 32494648

[19] M. Küß , M. Heigl , L. Flacke , A. Hörner , M. Weiler , A. Wixforth , M. Albrecht , Phys. Rev. Appl. 2021, 15, 034060. 10.1103/PhysRevLett.125.217203 33275006

[20] K. Wagner , A. Kákay , K. Schultheiss , A. Henschke , T. Sebastian , H. Schultheiss , Nat. Nanotechnol. 2016, 11, 432. 26828849 10.1038/nnano.2015.339

[21] F. Garcia‐Sanchez , P. Borys , R. Soucaille , J.‐P. Adam , R. L. Stamps , J.‐V. Kim , Phys. Rev. Lett. 2015, 114, 247206. 26197006 10.1103/PhysRevLett.114.247206

[22] J. M. Winter , Phys. Rev. 1961, 124, 452.

[23] V. Sluka , T. Schneider , R. A. Gallardo , A. Kákay , M. Weigand , T. Warnatz , R. Mattheis , A. Roldán‐Molina , P. Landeros , V. Tiberkevich , A. Slavin , G. Schütz , A. Erbe , A. Deac , J. Lindner , J. Raabe , J. Fassbender , S. Wintz , Nat. Nanotechnol. 2019, 14, 328. 30804478 10.1038/s41565-019-0383-4

[24] Y. Henry , O. Gladii , M. Bailleul , arXiv: 1611.06153 , 2016.

[25] Y. Henry , D. Stoeffler , J.‐V. Kim , M. Bailleul , Phys. Rev. B 2019, 100, 024416.

[26] J. Chen , J. Hu , H. Yu , IScience 2020, 23, 101153. 32450517 10.1016/j.isci.2020.101153 PMC7251948

[27] S. Wintz , V. Tiberkevich , M. Weigand , J. Raabe , J. Lindner , A. Erbe , A. Slavin , J. Fassbender , Nat. Nanotechnol. 2016, 11, 948. 27428277 10.1038/nnano.2016.117

[28] M. Baumgartner , K. Garello , J. Mendil , C. O. Avci , E. Grimaldi , C. Murer , J. Feng , M. Gabureac , C. Stamm , Y. Acremann , S. Finizio , S. Wintz , J. Raabe , P. Gambardella , Nat. Nanotechnol. 2017, 12, 980. 28825713 10.1038/nnano.2017.151

[29] S. Finizio , S. Wintz , D. Bracher , E. Kirk , A. S. Semisalova , J. Förster , K. Zeissler , T. Weßels , M. Weigand , K. Lenz , A. Kleibert , J. Raabe , Phys. Rev. B 2018, 98, 104415.

[30] S. Finizio , S. Wintz , K. Zeissler , A. V. Sadovnikov , S. Mayr , S. A. Nikitov , C. H. Marrows , J. Raabe , Nano Lett. 2019, 19, 375. 30517003 10.1021/acs.nanolett.8b04091

[31] E. Albisetti , S. Tacchi , R. Silvani , G. Scaramuzzi , S. Finizio , S. Wintz , C. Rinaldi , M. Cantoni , J. Raabe , G. Carlotti , R. Bertacco , E. Riedo , D. Petti , Adv. Mater. 2020, 32, 1906439. 10.1002/adma.201906439 31944413

[32] N. Träger , F. Lisiecki , R. Lawitzki , M. Weigand , H. Głowiński , G. Schütz , G. Schmitz , P. Kuświk , M. Krawczyk , J. Gräfe , P. Gruszecki , Phys. Rev. B 2021, 103, 014430. 10.1103/PhysRevLett.126.057201 33605763

[33] S. A. Montoya , S. Couture , J. J. Chess , J. C. T. Lee , N. Kent , M.‐Y. Im , S. D. Kevan , P. Fischer , B. J. McMorran , S. Roy , V. Lomakin , E. E. Fullerton , Phys. Rev. B 2017, 95, 224405.

[34] J. C. T. Lee , J. J. Chess , S. A. Montoya , X. Shi , N. Tamura , S. K. Mishra , P. Fischer , B. J. McMorran , S. K. Sinha , E. E. Fullerton , S. D. Kevan , S. Roy , Appl. Phys. Lett. 2016, 109, 022402.

[35] S. A. Montoya , S. Couture , J. J. Chess , J. C. T. Lee , N. Kent , D. Henze , S. K. Sinha , M.‐Y. Im , S. D. Kevan , P. Fischer , B. J. McMorran , V. Lomakin , S. Roy , E. E. Fullerton , Phys. Rev. B 2017, 95, 024415.

[36] S. A. Montoya , R. Tolley , I. Gilbert , S.‐G. Je , M.‐Y. Im , E. E. Fullerton , Phys. Rev. B 2018, 98, 104432.

[37] R. D. Desautels , L. DeBeer‐Schmitt , S. A. Montoya , J. A. Borchers , S.‐G. Je , N. Tang , M.‐Y. Im , M. R. Fitzsimmons , E. E. Fullerton , D. A. Gilbert , Phys. Rev. Mater. 2019, 3, 104406. 10.1103/physrevmaterials.3.081401 PMC11474935 39411750

[38] M. Heigl , S. Koraltan , M. Vaňatka , R. Kraft , C. Abert , C. Vogler , A. Semisalova , P. Che , A. Ullrich , T. Schmidt , J. Hintermayr , D. Grundler , M. Farle , M. Urbánek , D. Suess , M. Albrecht , Nat. Commun. 2021, 12, 2611. 33972515 10.1038/s41467-021-22600-7 PMC8110839

[39] J. Gräfe , M. Weigand , B. Van Waeyenberge , A. Gangwar , F. Groß , F. Lisiecki , J. Rychly , H. Stoll , N. Träger , J. Förster , F. Stobiecki , J. Dubowik , J. Klos , M. Krawczyk , C. H. Back , E. J. Goering , G. Schütz , Proc. SPIE 2019, 11090, 1109025.

[40] See Supporting Information including S1. MFM and FMR characterization of the Fe/Gd multilayers; S2. Influence on the magnetic textures from the periodicity and sizes of nanowire arrays on Fe/Gd samples; S3. Dispersion relations over the thickness of Fe/Gd multilayers in micromagnetic simulation; S4. Spin dynamics at f = 0.46 GHz and f = 0.58 GHz; S5. Spin dynamics at f = 0.38 GHz in the bare Fe/Gd multilayers; S6. Dispersion relations of spin waves in the domain walls with fixed Co magnetization in micromagnetic simulation; S7. Super‐domain structure and super‐domain boundary; S8. Programmable magnonic logic gates constructed by domain wall waveguides in Fe/Gd multilayers.

[41] A. Vansteenkiste , J. Leliaert , M. Dvornik , M. Helsen , F. Garcia‐Sanchez , B. Van Waeyenberge , AIP Adv. 2014, 4, 107133.

[42] J. Leliaert , J. Mulkers , J. De Clercq , A. Coene , M. Dvornik , B. Van Waeyenberge , AIP Adv. 2017, 7, 125010.

[43] H. Oezelt , L. Qu , A. Kovacs , J. Fischbacher , M. Gusenbauer , R. Beigelbeck , D. Praetorius , M. Yano , T. Shoji , A. Kato , R. Chantrell , M. Winklhofer , G. T. Zimanyi , T. Schrefl , npj Comput. Mater. 2022, 8, 35.

[44] G. Venkat , H. Fangohr , A. Prabhakar , J. Magn. Magn. Mater. 2018, 450, 34.

[45] G. Duerr , K. Thurner , J. Topp , R. Huber , D. Grundler , Phys. Rev. Lett. 2012, 108, 227202. 23003645 10.1103/PhysRevLett.108.227202

[46] L. Körber , R. Verba , J. A. Otálora , V. Kravchuk , J. Lindner , J. Fassbender , A. Kákay , Phys. Rev. B 2022, 106, 014405.

[47] E. Albisetti , D. Petti , G. Sala , R. Silvani , S. Tacchi , S. Finizio , S. Wintz , A. Calò , X. Zheng , J. Raabe , E. Riedo , R. Bertacco , Commun. Phys. 2018, 1, 56.

[48] L.‐J. Chang , J. Chen , D. Qu , L.‐Z. Tsai , Y.‐F. Liu , M.‐Y. Kao , J.‐Z. Liang , T.‐S. Wu , T.‐M. Chuang , H. Yu , S.‐F. Lee , Nano Lett. 2020, 20, 3140. 32323994 10.1021/acs.nanolett.9b05133

[49] Z. Li , B. Dong , Y, He , A. Chen , X. Li , J.‐H. Tian , C. Yan , Nano Lett. 2021, 21, 4708. 34014682 10.1021/acs.nanolett.1c00971

[50] K. Vogt , F. Y. Fradin , J. E. Pearson , T. Sebastian , S. D. Bader , B. Hillebrands , A. Hoffmann , H. Schultheiss , Nat. Commun. 2014, 5, 3727. 24759754 10.1038/ncomms4727

[51] Q. Wang , M. Kewenig , M. Schneider , R. Verba , F. Kohl , B. Heinz , M. Geilen , M. Mohseni , B. Lägel , F. Ciubotaru , C. Adelmann , C. Dubs , S. D. Cotofana , O. V. Dobrovolskiy , T. Brächer , P. Pirro , A. V. Chumak , Nat. Electron. 2020, 3, 765.

[52] B. Heinz , T. Brächer , M. Schneider , Q. Wang , B. Lägel , A. M. Friedel , D. Breitbach , S. Steinert , T. Meyer , M. Kewenig , C. Dubs , P. Pirro , A. V. Chumak , Nano Lett. 2020, 20, 4220. 32329620 10.1021/acs.nanolett.0c00657 PMC7291357

[53] M. Jamali , J. H. Kwon , S.‐M. Seo , K.‐J. Lee , H. Yang , Sci. Rep. 2013, 3, 3160. 24196318 10.1038/srep03160 PMC3819604

[54] S. Seki , M. Garst , J. Waizner , R. Takagi , N. D. Khanh , Y. Okamura , K. Kondou , F. Kagawa , Y. Otani , Y. Tokura , Nat. Commun. 2020, 11, 256. 31937762 10.1038/s41467-019-14095-0 PMC6959257

[55] X. Liang , Z. Wang , P. Yang , Y. Zhou , Phys. Rev. B 2022, 106, 224413.

[56] F. Millo , J.‐P. Adam , C. Chappert , J.‐V. Kim , A. Mouhoub , A. Solignac , T. Devolder , Phys. Rev. Applied 2023, 20, 054051.

[57] R. Gallardo , M. Weigand , K. Schultheiss , A. Kakay , R. Mattheis , J. Raabe , G. Schütz , A. Deac , J. Lindner , S. Wintz , ACS Nano 2024, 18, 5249. 38314709 10.1021/acsnano.3c08390 PMC10883124

[58] G. Schütz , W. Wagner , W. Wilhelm , P. Kienle , R. Zeller , R. Frahm , G. Materlik , Phys. Rev. Lett. 1987, 58, 737. 10035022 10.1103/PhysRevLett.58.737

[59] M. Weigand , S. Wintz , J. Gräfe , M. Noske , H. Stoll , B. Van Waeyenberge G. Schütz , Crystals 2022, 12, 1029.

[60] S. Mayr , J. Förster , S. Finizio , K. Schultheiss , R. A. Gallardo , R. Narkovicz , G. Dieterle , A. Semisalova , J. Bailey , E. Kirk , A. Suszka , J. Lindner , J. Gräfe , J. Raabe , G. Schütz , M. Weigand , H. Stoll , S. Wintz , Appl. Phys. Rev. 2024, 11, 041411.

[61] F. Groß , N. Träger , J. Gräfe , SoftwareX 2021, 15, 100705.

[62] M. B. Hahn , J. Phys. Commun. 2019, 3, 075009.

