# Supplementary material for: Nonreciprocal Spin Waves in Nanoscale Hybrid Néel–Bloch–Néel Domain Walls Detected by Scanning X‐Ray Microscopy in Perpendicular Magnetic Anisotropic Fe/Gd Multilayers
Source: Adv Mater. 2025 Aug 4;37(40):e08181. doi: 10.1002/adma.202508181 (PMC12510291; doi:10.1002/adma.202508181)
Supplement: Supplementary file 1 — Supporting Information [file ADMA-37-e08181-s003.pdf]

ADVANCED MATERIALS

Supporting Information

for *Adv. Mater.*, DOI 10.1002/adma.202508181

Nonreciprocal Spin Waves in Nanoscale Hybrid Néel–Bloch–Néel Domain Walls Detected by Scanning X-Ray Microscopy in Perpendicular Magnetic Anisotropic Fe/Gd Multilayers

Ping Che, Axel J. M. Deenen, Andrea Mucchietto, Joachim Gräfe, Michael Heigl, Korbinian Baumgaertl, Markus Weigand, Michael Bechtel, Sabri Koraltan, Gisela Schütz, Dieter Suess, Manfred Albrecht and Dirk Grundler**

Supplementary Materials for Nonreciprocal Spin Waves in Nanoscale Hybrid Néel-Bloch-Néel Domain Walls Detected by Scanning X-ray Microscopy in Perpendicular Magnetic Anisotropic Fe/Gd Multilayers

Ping Che,[1,*] Axel Deenen,[1] Andrea Mucchietto,[1] Joachim Gräfe,[2] Michael Heigl,[3]
Korbinian Baumgaertl,[1] Markus Weigand,[4] Michael Bechtel,[4] Sabri Koraltan,[5,6]
Gisela Schütz,[2] Dieter Suess,[5,7] Manfred Albrecht,[3] and Dirk Grundler[1,8,†]

[1]*Laboratory of Nanoscale Magnetic Materials and Magnonics, Institute of Materials (IMX), École Polytechnique Fédérale de Lausanne (EPFL), 1015 Lausanne, Switzerland*
[2]*Max Planck Institute for Intelligent Systems, Heisenbergstraße 3, 70569 Stuttgart, Germany*
[3]*Institute of Physics, University of Augsburg, Universitätsstrasse 1, D-86159 Augsburg, Germany*
[4]*Helmholtz-Zentrum Berlin für Materialien und Energie, Albert-Einstein-Straße 15, 12489 Berlin, Germany*
[5]*Physics of Functional Materials, Faculty of Physics, University of Vienna, Vienna, Austria*
[6]*Vienna Doctoral School in Physics, University of Vienna, Vienna, Austria*
[7]*Research Platform MMM Mathematics-Magnetism-Materials, University of Vienna, Vienna, Austria*
[8]*Institute of Electrical and Micro Engineering (IEL), École Polytechnique Fédérale de Lausanne (EPFL), 1015 Lausanne, Switzerland*

Contents

S1. MFM and FMR characterization of the Fe/Gd multilayers 2

S2. Influence on the magnetic textures from the periodicity and sizes of nanowire arrays on Fe/Gd samples 3

S3. Dispersion relations over the thickness of Fe/Gd multilayers in micromagnetic simulation 5

S4. Spin dynamics at $f = 0.46$ GHz and $f = 0.58$ GHz 6

S5. Spin dynamics at $f = 0.38$ GHz in the bare Fe/Gd multilayers 7

S6. Dispersion relations of spin waves in the domain walls with fixed Co magnetization in micromagnetic simulation 8

S7. Super-domain structure and super-domain boundary 9

S8. Programmable magnonic logic gates constructed by domain wall waveguides in Fe/Gd multilayers 10

References 11

* ping.che@epfl.ch; Present address: Laboratoire Albert Fert, CNRS, Thales, Université Paris-Saclay, Palaiseau 91767, France.
† dirk.grundler@epfl.ch

S1. MFM and FMR characterization of the Fe/Gd multilayers

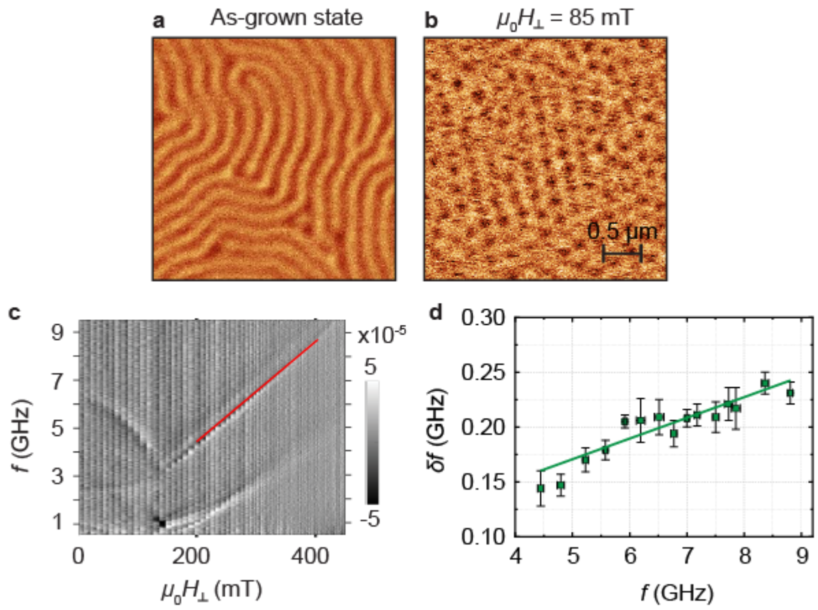

Figure S1. a) and b) MFM imaging of the as-grown state of stripe-shape domains and of the dipole skyrmion lattice states when the external field $\mu_0 H_\perp$ swept from 0 mT to 85 mT. c) FMR spectra ΔS_{21} of Pt/[Fe (0.5 nm)/Gd (0.35 nm)]$_{80}$/Pt multilayers grown on Si substrate. Red line is the fitted Kittel formula. d) Linewidth of main resonance mode (green squares). The green line is the linear fit with Equ. S1 for damping parameter extraction.

Magnetic resonance spectra of transmission signal ΔS_{21} with external field $\mu_0 H_\perp$ swept from 0 to 450 mT after saturated at -450 mT are plotted in Fig. S1 c). Above $\mu_0 H_\perp = 147$ mT, the multilayer exhibits a resonance frequency increase with magnetic field strength, which is the sign of the saturated state. The mode between 3 GHz to 9 GHz is the main mode, as the other mode below 4 GHz vanishes when the field strength is increased. The main mode was analyzed for anisotropy strength, saturation magnetization and Gilbert damping parameters. The experimental data was fitted using the Kittel formula: $f = \frac{|\gamma|}{2\pi}(H_\perp + 4\pi M_{\text{eff}})$ [1], where γ is the gyromagnetic ratio and $4\pi M_{\text{eff}} = 4\pi M_{\text{S}} - 2K_{\text{U}}/M_{\text{S}}$ is the effective magnetization consisting of both shape anisotropy and perpendicular magnetic anisotropy. From the slope and interpretation of the fitting, $\gamma = 2\pi \times (21.1 \pm 0.1)$ and $4\pi M_{\text{eff}} = (-14 \pm 10)$ mT were extracted. Combined with the $\mu_0 M_{\text{S}} = 319.9 \pm 1.0$ emu/cm^3 extracted from the hysteresis measurement, the anisotropy strength can be calculated $K_{\text{U}} = 66.2$ kJ/m^3. The full width at half maximum (FWHM) δf of the fitted peaks is shown in Fig.S1 d) with standard errors from Lorentz peak function fittings. To extract the damping parameter α, the frequency dependent δf variation was fitted linearly by the equation[2]:

$$\delta f = \frac{|\gamma|}{2\pi}\mu_0 \Delta H + 2\alpha f \tag{S1}$$

Here, μ_0 is the permeability of free space. The damping parameter was interpreted to be $\alpha = 0.009 \pm 0.001$, which is low compared with interfical DMI systems hosting skyrmions and consistent with the reported values from the same multilayer systems[3].

S2. Influence on the magnetic textures from the periodicity and sizes of nanowire arrays on Fe/Gd samples

TABLE S1. Table of periodicity and sizes of nanowire arrays on Fe/Gd samples.

Device No.	Width of the Co nanowire w_{nw} (nm)	Periodicity p_{nw} (nm)
1	150	300
2	175	350
3	200	400
4	225	450
5	250	500

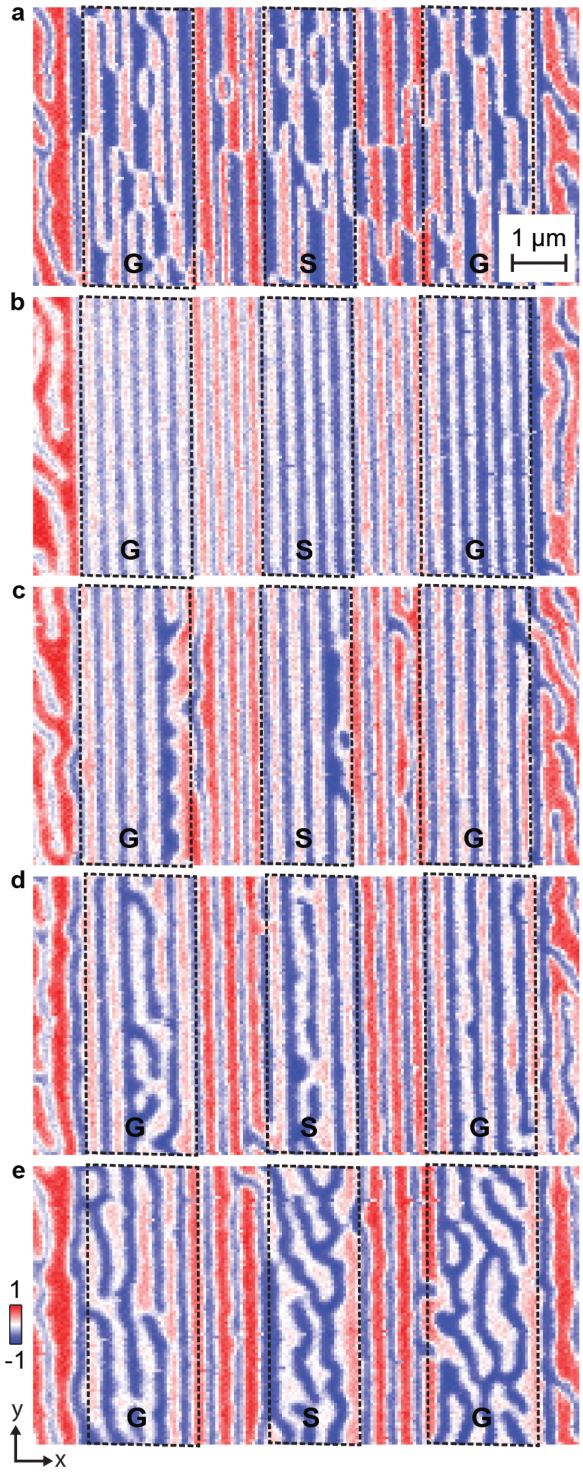

Figure S2. Static STXM images of domains in sample-B with nanowire arrays at $\mu_0 H_\perp = 0$ mT corresponding to sample No. 1 to sample No. 5 (a) to e)). The color bar represents normalized X-ray transmission intensity. Dashed black frames indicate the regions of the CPW lines.

S3. Dispersion relations over the thickness of Fe/Gd multilayers in micromagnetic simulation

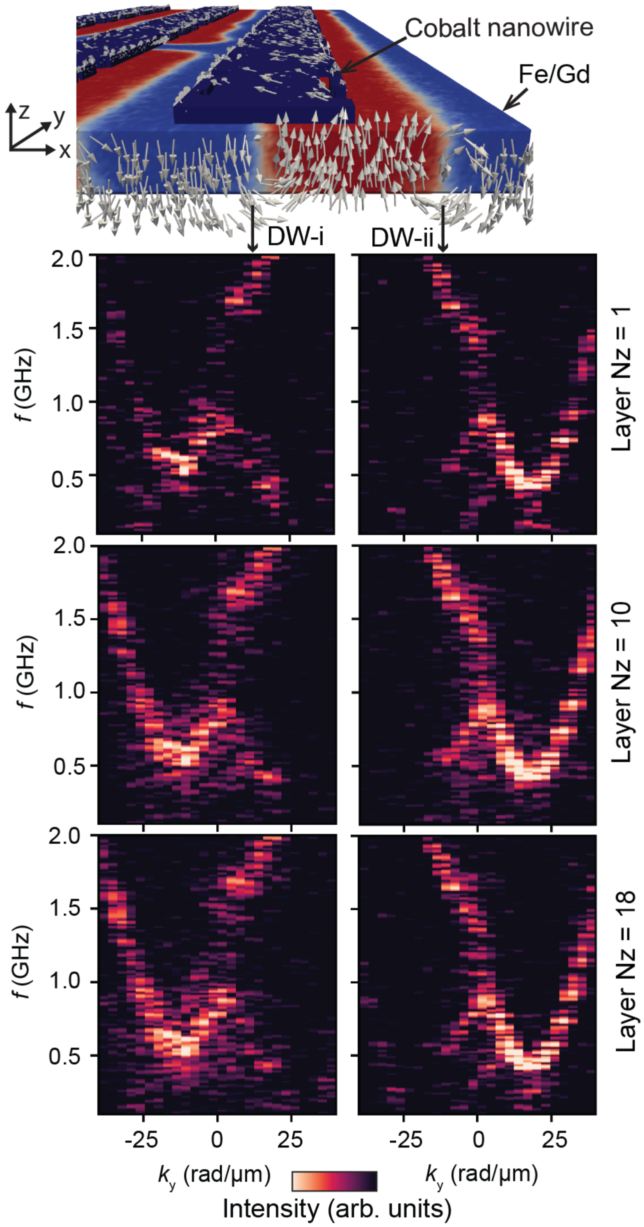

Figure S3. Dispersion relations over the thickness of Fe/Gd multilayers in micromagnetic simulation, in the two type of DWs in the domain configuration. The middle row of Layer $N_z = 10$ is reprinted from Fig. 1 e) and f) for direct comparison.

S4. Spin dynamics at $f = 0.46$ GHz and $f = 0.58$ GHz

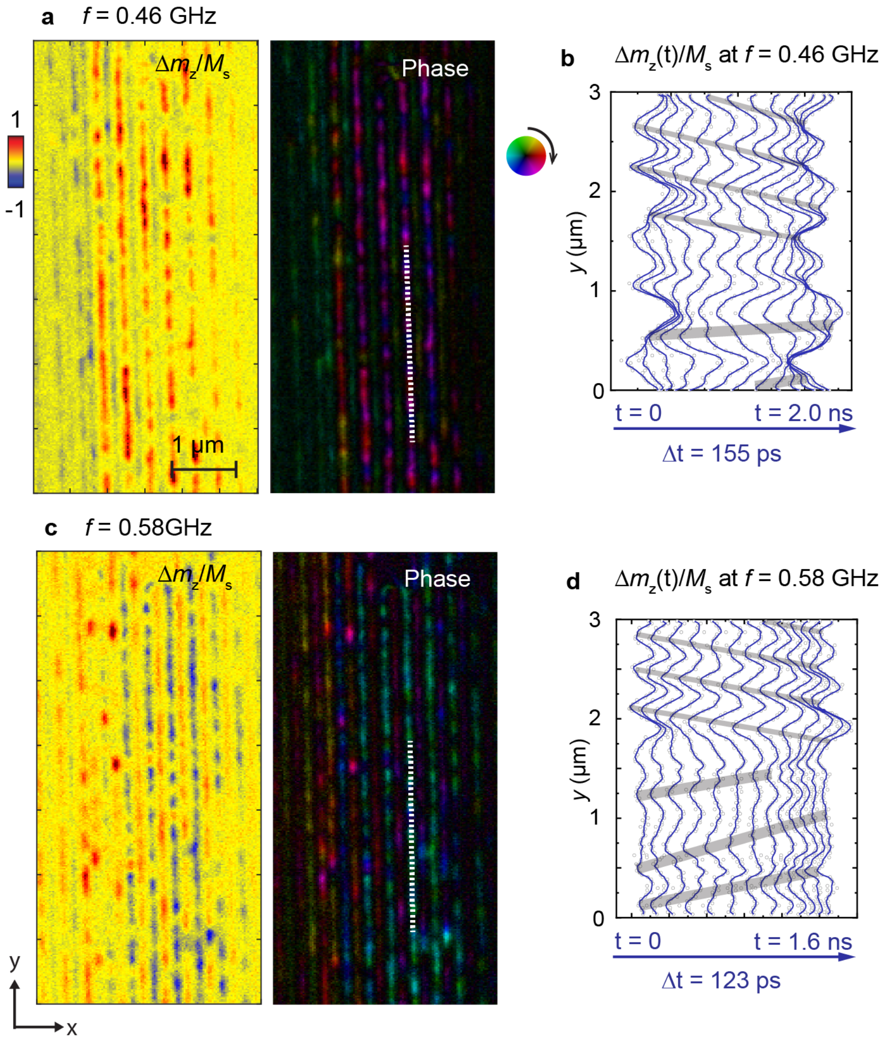

Figure S4. a) and c) Snapshot of the spin dynamics and their phase (integrated with the amplitude) at $f = 0.46$ GHz and $f = 0.58$ GHz normalized to the static image. b) and d) The time evolution of the dynamics components of the transmission signal taken from the region marked by the dashed white lines in all the phase images. Grey shadows are the eye-guide by marking the peaks moving with time.

S5. Spin dynamics at $f = 0.38$ GHz in the bare Fe/Gd multilayers

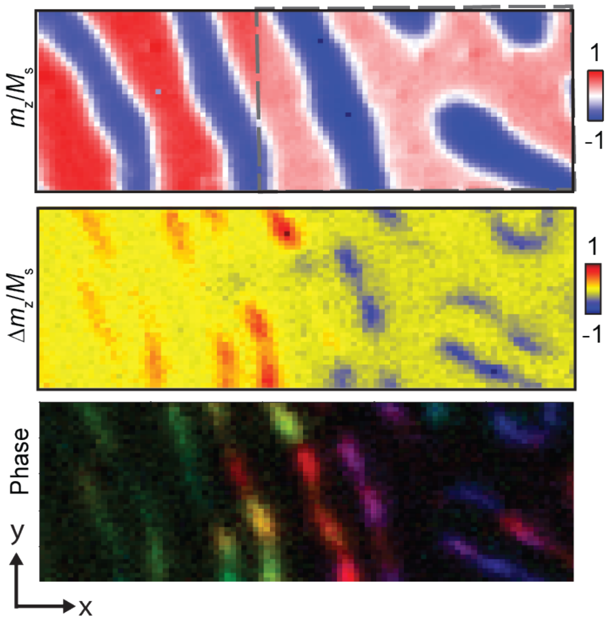

Figure S5. a) Static STXM images of domains where the spin dynamics are investigated. Grey frames indicated the region covered by the signal line. b) Snapshot of the spin dynamics at $f = 0.31$ GHz normalized to the static image in a). c) Phase information of the spin dynamics.

S6. Dispersion relations of spin waves in the domain walls with fixed Co magnetization in micromagnetic simulation

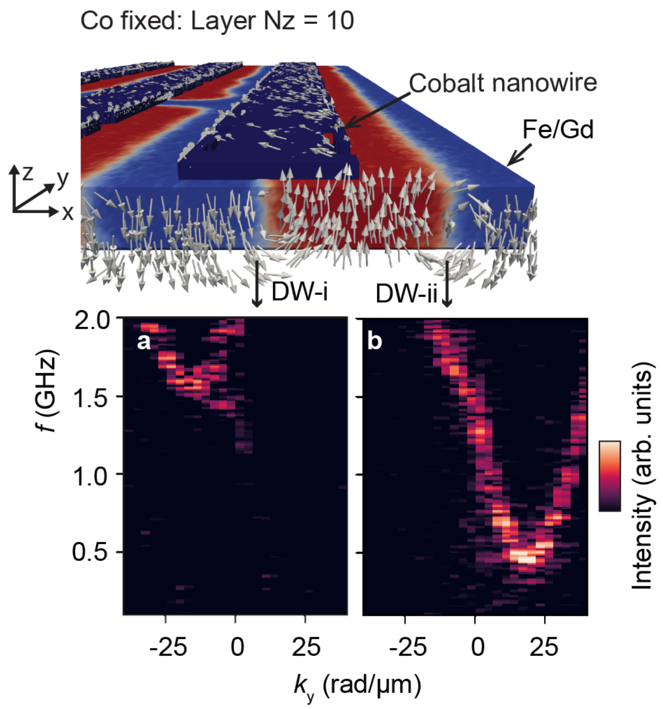

Figure S6. Dispersion relations over the thickness of Fe/Gd multilayers in micromagnetic simulation, in the two type of DWs in the domain configuration with fixed Co magnetization.

S7. Super-domain structure and super-domain boundary

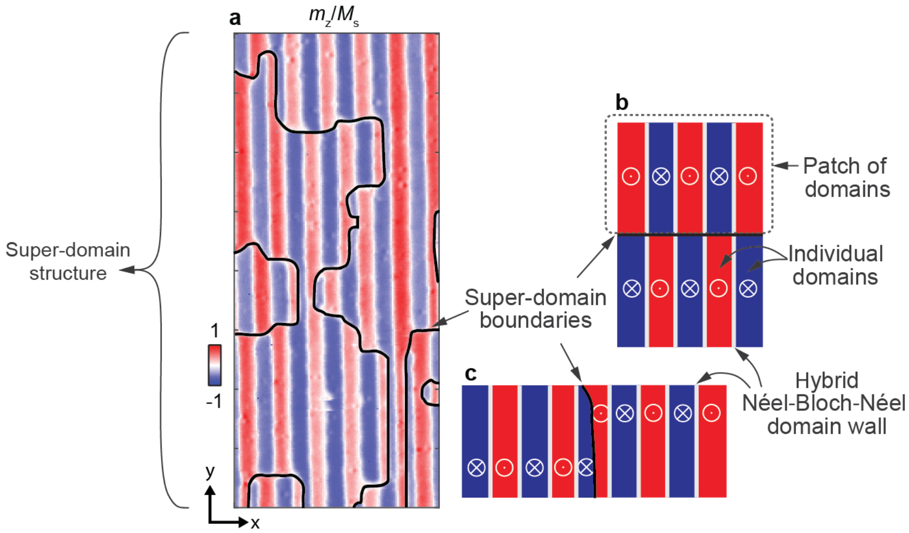

Figure S7. (a) Super-domain structures. (b) and (c) Schematic of the two type of configuration in the super-domain structure.

S8. Programmable magnonic logic gates constructed by domain wall waveguides in Fe/Gd multilayers

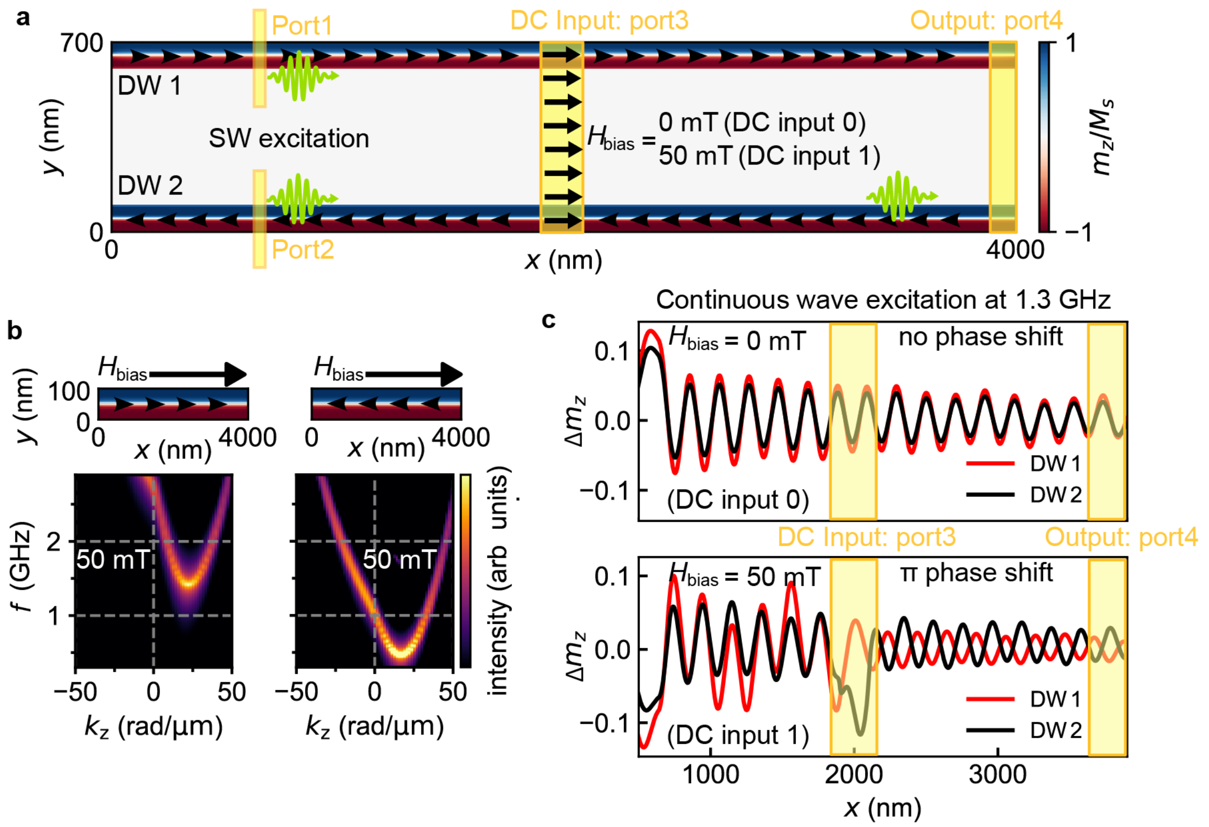

d

NOT gate

Input (Port3)	Output (Port4)
0	1
1	0

OR gate (DC input 0)

Input1 (Port1)	Input2 (Port2)	Output (Port4)
1	1	1
1	0	1
0	1	1
0	0	0

XOR gate (DC input 1)

Input1 (Port1)	Input2 (Port2)	Output (Port4)
1	1	0
1	0	1
0	1	1
0	0	0

Figure S8. a) Illustration of the logic gates constructed by two domain walls (DW) in Fe/Gd multilayers in micromagnetic simulation. RF currents are injected in region of port1 and port2 for spin waves (SW) excitation at $f = 1.3$ GHz. A DC current is applied at region of port3 to tune the dispersion relation. Here, when the current is on, a bias field $H_{\text{bias}} = 50$ mT is applied locally in the region of the port3. At the end of **x**-axis, an antenna is used to collect the SW signal at the resonance frequency $f = 1.3$ GHz. Blue and red color represents the **z** components of the magnetization and the black arrow between the domains indicate the in-plane component of the magnetization of the DWs. b) Numerically computed spin wave dispersion relations of a 100 wide waveguide with a DW when a uniform field of 50 mT along the positive **x**-direction is applied over the entire waveguide. The cases where the in-plane magnetization of the Bloch-like DW is along the positive and negative **x**-direction are considered. c) The dynamic magnetization component along **z**-axis, indicating the profile of the propagating spin waves along the **x**-axis. In the upper panel, DC input is zero and there is no H_{bias} applied. The constructive interference happens at the Output: port4. In the lower panel, DC input is on and $H_{\text{bias}} = 50$ mT is applied. The destructive interference happens at the Output: port4. d) Logic gates constructed by combination of different ports indicated in a).

Magnonic logic gates are constructed using two DWs in bare 68 nm thick Fe/Gd multilayers, as illustrated in Fig. S8a). Each DW is located in a 100 nm wide waveguide. The DWs under consideration have identical out-of-plane configurations (up/down) but the Bloch-like DWs have opposite in-plane magnetization (along +x and -x for DW 1

and 2, respectively). The nonreciprocal spin-wave dispersion relation is as discussed in the main text. Spin waves are excited in both DWs at $f = 1.3$ GHz by injecting radio-frequency (RF) currents at Port1 and Port2. When there is no DC current applied at Port3, there is no in-plane bias field, H_{bias}. This state is labeled as DC input 0. Under this configuration, the dispersion relations of spin waves in DW1 and DW2 are identical, leading to constructive interference at the region of Output: Port4. When a DC current is applied at Port3 (labeled as DC input 1), a local bias field is generated. Here, we only consider an in-plane bias field $H_{\text{bias}} = 50$ mT directed along the positive x-direction over a region of 200 nm wide. Due to the antiparallel magnetization of DW1 and DW2, the dispersion relations of the two DWs differ (Fig. S8b)). In this case, spin waves with a π phase shift arrive at Output: Port4, resulting in destructive interference.

Using the design in Fig. S8a), three types of logic gates can be constructed:

1. NOT gate: The DC input at Port3 serves as the input, while Port4 is the output. Spin wave excitation in DW1 and DW2 is always active. When the DC input is 0, constructive interference occurs, and the output is 1. When the DC input is 1, destructive interference occurs, and the output is 0.

2. OR gate: With the DC input fixed at 0, the RF currents at Port1 and Port2 act as the two inputs, and Port4 serves as the output. The output is defined as 1 if a nonzero spin-wave amplitude is detected at Port4. Therefore, the output is 0 only when both inputs are zero.

3. XOR gate: With the DC input fixed at 1, the RF currents at Port1 and Port2 act as the two inputs, and Port4 serves as the output. The output is defined as 1 if a nonzero spin-wave amplitude is detected at Port4. When spin waves are excited simultaneously at both input ports, destructive interference leads to a zero output, as does the absence of input at both ports.

---

[1] H. Głowiński, A. Zywczak, J. Wrona, A. Krysztofik, I. Gościańska, T. Stobiecki, and J. Dubowik, *J. Phys.: Condens.* Matter **2017**, 29, 485803.

[2] H. Yu, R. Huber, T. Schwarze, F. Brandl, T. Rapp, P. Berberich, G. Duerr, and D. Grundler, *Appl. Phys. Lett.* **2012**, 100, 262412.

[3] S. A. Montoya, S. Couture, J. J. Chess, J. C. T. Lee, N. Kent, M.-Y. Im, S. D. Kevan, P. Fischer, B. J. McMorran, S. Roy, V. Lomakin, and E. E. Fullerton, *Phys. Rev. B* **2017**, 95, 224405.

[4] J. Leliaert, J. Mulkers, J. De Clercq, A. Coene, M. Dvornik, B. Van Waeyenberge, *AIP Adv.* **2017**, 7, 125010.

[5] H. Oezelt, L. Qu, A. Kovacs, J. Fischbacher, M. Gusenbauer, R. Beigelbeck, D. Praetorius, M. Yano, T. Shoji, A. Kato, R. Chantrell, M. Winklhofer, G. T. Zimanyi, T. Schrefl, *Npj Comput. Mater.* **2022**, 8, 35.
