# Supplementary figures and images for: Nonreciprocal Spin Waves in Nanoscale Hybrid Néel–Bloch–Néel Domain Walls Detected by Scanning X‐Ray Microscopy in Perpendicular Magnetic Anisotropic Fe/Gd Multilayers
Source: Adv Mater. 2025 Aug 4;37(40):e08181. doi: 10.1002/adma.202508181 (PMC12510291; doi:10.1002/adma.202508181)

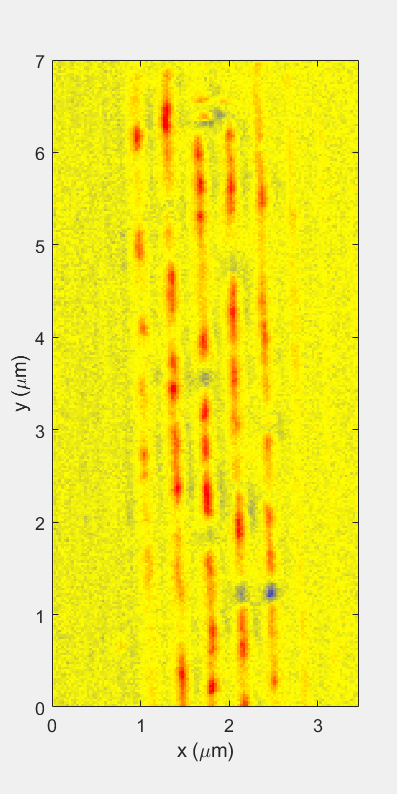

Supplement: Supplementary file 2 — Supplemental Movie 1 [file ADMA-37-e08181-s001.gif]

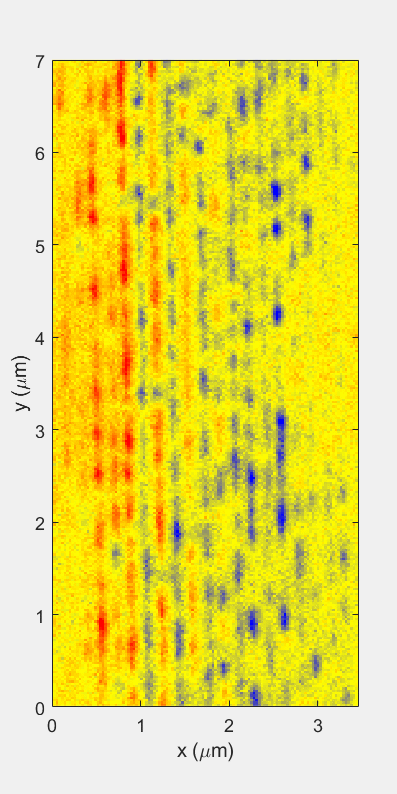

Supplement: Supplementary file 3 — Supplemental Movie 2 [file ADMA-37-e08181-s002.gif]
